# MTH1 Inhibition Alleviates Immune Suppression and Enhances the Efficacy of Anti-PD-L1 Immunotherapy in Experimental Mesothelioma

**DOI:** 10.3390/cancers15204962

**Published:** 2023-10-12

**Authors:** Sophia F. Magkouta, Photene C. Vaitsi, Marianthi P. Iliopoulou, Apostolos G. Pappas, Chrysavgi N. Kosti, Katherina Psarra, Ioannis T. Kalomenidis

**Affiliations:** 1 “Marianthi Simou Laboratory”, 1st Department of Critical Care and Pulmonary Medicine, Evangelismos Hospital, School of Medicine, National and Kapodistrian University of Athens 10676 Athens, Greece; photenevaitsi@gmail.com (P.C.V.); dr.mar.iliopoulou@gmail.com (M.P.I.); apo.pappas88@gmail.com (A.G.P.); xkdocxk@gmail.com (C.N.K.); ikalom@med.uoa.gr (I.T.K.); 2 Department of Immunology-Histocompatibility, Evangelismos Hospital, 10675 Athens, Greece; katherina@kyttarometria.gr

**Keywords:** mesothelioma, MTH1, immunotherapy

## Abstract

**Simple Summary:**

The role of MTH1 in the tumor-related immune responses in mesothelioma remains unknown. We hypothesized that MTH1 regulates immune responses and enhances the efficacy of anti-PD-L1 immunotherapy. We focused on central immune players such as tumor-associated macrophages. MTH1 inhibition enhances M1 macrophage polarization, stimulates CD8 fitness and promotes the activation of DC. Combining anti-PD-L1 immunotherapy with MTH1 inhibitor impairs mesothelioma tumor growth and mesothelioma-associated pleural effusion accumulation more effectively compared to each monotherapy.

**Abstract:**

Background: MTH1 protects tumor cells and their supporting endothelium from lethal DNA damage triggered by oxidative stress in the tumor microenvironment, thus promoting tumor growth. The impact of MTH1 on the tumor-related immune compartment remains unknown. We hypothesized that MTH1 regulates immune fitness and therefore enhances the activity of currently used immunotherapeutic regimens. Methods: Our hypotheses were validated in two syngeneic murine mesothelioma models using the clinically relevant MTH1 inhibitor, karonudib. We also examined the effect of combined MTH1 and PD-L1 blockade in mesothelioma progression, focusing on the main immune players. Results: Karonudib administration enhances M1 macrophage polarization, stimulates CD8 expansion and promotes the activation of DC and T cells. Combined administration of PD-L1 and MTH1 inhibitors impairs mesothelioma tumor growth and mesothelioma-associated pleural effusion accumulation more effectively compared to each monotherapy. Conclusions: Combined MTH1 and PD-L1 inhibition holds promise for the successful clinical management of mesothelioma.

## 1. Introduction

Malignant pleural mesothelioma is the most common primary tumor of the pleural cavity with an increasing global incidence [1] and dismal prognosis [2]. Current conventional chemotherapy regimens present moderate survival benefit [2,3]. Notably, immunotherapy has been recently approved as the first-line treatment in mesothelioma [4,5], providing a significant improvement in patients’ overall survival versus conventional therapy, and should be considered as a new standard of care [5]. These evolutions fuel the interest in deeply investigating mesothelioma immune microenvironment and its interactions with mesothelioma cells in order to devise more potent immune-based anti-mesothelioma therapies.

Elevated tumor immunogenicity is commonly the result of increased intrinsic genetic instability [6], which leads to a broadened neoantigen repertoire presentation (which can activate the adaptive immune response) or the accumulation of cytosolic DNA (which can activate the innate immune response) [7,8]. This observation can be therapeutically exploited since it provides us with a strong rationale for combining immunotherapies with DNA-damage-enhancing agents. Novel agents targeting DNA repair proteins have given promising results alone or combined with anti-PD-L1 immunotherapy [9,10,11]. However, data are limited regarding the interactions between DNA repair targeting and response to immunotherapy or its immune-activating properties. Whereas the role of DNA damage and repair in tumor cells’ phenotype has been studied in recent years, the importance of DNA damage in non-malignant cells of the tumor microenvironment remains unknown.

We have recently underlined the importance of MTH1 for mesothelioma cell survival and tumor progression [12]. This enzyme prevents the incorporation of oxidized nucleotides in DNA during replication, which would cause DNA damage and lead to cell death [13]. Notably, MTH1 is vital for the survival of tumor cells but nonessential for normal ones [13] and therefore presents an attracting target for cancer therapy. The most potent inhibitor of this enzyme, karonudib (TH1579), is currently already under phase I clinical testing (Clinicaltrial.gov, NCT03036228). We recently showed that apart from halting mesothelioma progression, MTH1 inhibition impedes tumor angiogenesis by selectively targeting the tumor endothelium [12].

In the present study, we assumed that besides mesothelioma cells, MTH1 upregulation would be important for tumor-associated inflammatory cell survival. We hypothesized that MTH1 inhibition would affect tumor-associated inflammatory cell survival to modulate the tumor immune milieu and enhance the efficacy of immunotherapy by alleviating immune suppression.

## 2. Materials and Methods

### 2.1. Cell Lines and Reagents

Murine AE17 and AB1 mesothelioma cell lines were provided by Dr. YCG Lee (Centre for Asthma, Allergy, and Respiratory Research, School of Medicine and Pharmacology, University of Western Australia, Perth, Western Australia). Both cell lines were maintained in Dulbecco’s Modified Eagle’s Medium (DMEM) (10% fetal bovine serum). Bone-marrow-derived macrophages (BMDMs) were generated following standard protocols [14]. Briefly, bone marrow cells were flushed away from the cavity of murine femurs using a 10 mL syringe containing DMEM. Cells were then collected and resuspended in DMEM containing 20 ng/mL murine macrophage colony-stimulating factor (M-CSF) and cultured for seven days.

An MTH1 inhibitor, karonudib (Karolinska Nudt1 inhibitor, or else TH1579), was developed and kindly provided by Prof. Thomas Helleday (Division of Translational Medicine and Chemical Biology Karolinska Institutet, SciLifeLab, Stockholm, Sweden). Karonudib was formulated with hydroxypropyl-β-cyclodextrin (HPβCD, 10% *w*/*v*) (AppliChem GmbH, Darmstadt, Germany) in acetate buffer (pH = 4.5) and was freshly prepared for each administration. Hydroxy chloroquine sulfate (TLR-9 inhibitor) was purchased from Merck (Darmstadt, Germany). Murine anti-PD-L1 (Anti-PD-L1-mIgG1e3 InvivoFit™) and respective isotype control (mIgG1e3 InvivoFit™) were obtained by InvivoGen (Toulouse, France).

### 2.2. In Vivo Studies

C57BL/6 and Balb/c mice were purchased from BSRC Al. Fleming (Vari, Greece) and housed at the Animal Model Research Unit of Evangelismos Hospital. Mice received food and water ad libitum.

AE17 or AB1 (5 × 10^5^) mesothelioma cells were intrapleurally injected into 8–10-week-old C57BL/6 or Balb/c syngeneic mice, respectively [15]. Four days following tumor cell implantation, animals were divided into two groups (8 mice/group in AE17 tumor-bearing C57BL6, 5 mice/group in AB1 tumor-bearing Balb/c mice), receiving either karonudib (90 mg/kg body weight) or vehicle (10% HPβCD) twice a day, every other day, p.o (oral gavage). In experiments evaluating the potential benefits of karonudib on the efficacy of immunotherapy, mesothelioma-bearing mice were split into 4 groups (5–8 mice per group): control group (receiving vehicle and IgG), karonudib group (receiving karonudib and IgG), anti-PD-L1 group (receiving vehicle and anti-PD-L1) and karonudib + anti-PD-L1 group. Karonudib administration commenced on day 4, while anti-PD-L1 commenced on day 5 upon tumor cell inoculation.

In all cases, animals were euthanized 12–14 days after pleural injection of mesothelioma cells. Mesothelioma tumors were collected and weighed, while pleural fluid was retrieved and quantified.

### 2.3. Flow Cytometry

Immune cells isolated from pleural tumors and fluid were fixed, permeabilized and stained with anti-CD45 (30-F11), CD11b (M1/70), F4/80 (BM/8), Ly6C (HK1.4), Ly6G (RB6-8C5), IL10 (JES5-16E3), IL12 (C15.6), CD11c (HL3), MHCII (M5/114.15.2), CD3 (145-2C11), CD4 (GK1.5), CD8 (YTS1567.7), Foxp3 (MF14), Ki-67 (16A8), granzyme-B (QA18A28) and PD1 (29F.1A12) (all obtained from Biolegend, San Diego, CA, USA). Forward and side scatter profiles and CD45-positive staining were applied in order to select inflammatory populations. Macrophage populations were subsequently characterized as CD11b+/Ly6G-/Ly6Clow/F4/80+, M-MDSCs as CD11b+/Ly6G-/Ly6C, PMN-MDSCs as CD11b+/Ly6G+/Ly6Clow and activated DCs as CD11c+/MHCII+ cells. M1/M2 phenotypes were determined based on their IL-12/IL-10 expression ratio. Total CD3+/CD4+ and CD3+/CD8+ lymphocyte populations were also quantified. T-regulatory cells were determined as CD4+/ Foxp3+ cells, while activated CD8+ T-cells were identified by granzyme-B expression. Analysis was performed using the BD FACSCantoII flow cytometer and acquired data were analyzed by FlowJo Software V10 (LLC, Ashland, OR, USA).

### 2.4. In Vitro Studies

#### 2.4.1. Real-Time PCR

Quantification of MTH1 mRNA expression levels was performed by real-time PCR. Macrophages of tumor, pleural fluid or naïve pleural space were isolated using anti-F4/80-loaded (clone BM8, Biolegend, San Diego, CA, USA) streptavidin magnetic beads (MojoSort Streptavidin Nanobeads, Biolegend, San Diego, CA, USA) as previously reported [16]. Total mRNA of normal, pleural fluid and tumoral macrophages was isolated using the Nucleospin RNAplus kit (Macherey-Nagel, Düren, Germany). cDNA was subsequently prepared using the PrimeScript 1st strand cDNA Synthesis kit (Takara, Clontech, Mountain View, CA, USA). MTH1 mRNA levels were normalized to Gapdh gene expression as previously described [13].

#### 2.4.2. Western Blotting

BMDMs generated as aforementioned were seeded onto 6-well plates at a density of 2 × 105 cells/well and serum starved overnight. Cell-free DNA was secreted by AE17, or AE17 cells treated with karonudib (10 μM, overnight) isolated from culture supernatants using a commercial kit (Nucleospin, Macherey-Nagel Düren, Germany). BMDMs were subsequently treated with 20 ng/mL extracellular DNA for 2 h. TLR9 inhibitor chloroquine (Merck, Darmstadt, Germany) was used at 2 μg/mL for 40 min before addition of cfDNAs. Cell lysates were prepared and analyzed by Western blotting for phospho-p65 NFKB (#3031), total p65 NFKB (#4764), phospho-ikBa (#2859) and total-ikBa (#4812) (Cell Signaling Technology Inc., Danvers, MA, USA). Results were normalized to actin (#4970, Cell Signaling Technology Inc., Danvers, MA, USA). Visualized bands were quantified by GelPro Analyzer 6.3 (Media Cybernetics, L.P., Rockville, MD, USA)

### 2.5. Statistics

All values are presented as the mean ± standard error of mean (SEM). Differences between groups were evaluated using the 2-tailed Student’s *t*-test, or one-way analysis of variance (ANOVA) with Bonferroni post hoc test for multiple comparisons, as appropriate. *p* values less than 0.05 were considered statistically significant. Statistical analysis was performed using the Statistical Package for the Social Sciences v.13.0.0 (IMB, Armonk, NY, USA).

### 2.6. Study Approval

Experiments were approved by the Veterinary Administration Bureau, Prefecture of Athens, Greece (Decision No: 8/2019, 15 November 2019), under compliance with the national law and EU Directives.

## 3. Results

### 3.1. Μth-1 Inhibition Affects Tumor Macrophage Polarization through Extracellular DNA/TLR9/NFkB Signaling, and Enhances CD8 Infiltration and Activation and Promotes DC MHCII Expression

**Figure 1:**
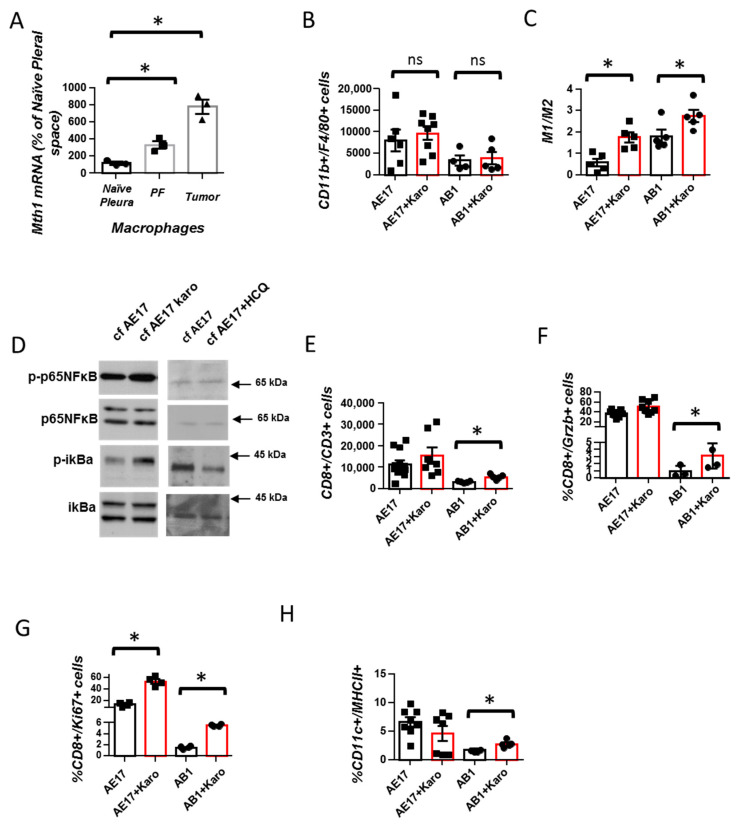
Μth-1 inhibition affects tumor macrophage polarization through extracellular DNA/TLR9/NFkB signaling, enhances CD8 infiltration and activation and promotes DC MHCII expression. (**A**) MTH1 mRNA expression levels of murine macrophages from normal pleural space, mesothelioma tumors and accompanying pleural fluid (PF) were determined by real-time PCR. Data presented as mean ± SEM, *n* = 3, * *p* < 0.05 compared to ‘naive’ macrophages, ns: not significant (**B**,**C**) AB1 and AE17 cells were intrapleurally injected into syngeneic Balb/c and C57Bl/6 mice, respectively. Mice received vehicle or karonudib (90 mg/kg body weight) twice bi-daily. Fourteen days later, mice were sacrificed, and tumors of vehicle- and karonudib-treated animals were analyzed by flow cytometry for total macrophage (CD11b+ F4/80+) populations and their IL12/IL10 expression ratio (indicative of M1/M2 polarization). Data are presented as mean ± SEM, *n* = 5–8, * *p* < 0.05 compared to vehicle. (**D**) Serum-starved bone-marrow-derived macrophages (BMDMs) were treated for 4 h with 30 ng cell-free DNA (CfDNA) secreted from AE17 cells pretreated with vehicle or karonudib (left panels). Alternatively, BMDMs were treated with TLR9 inhibitor HCQ (2 μg/mL) for 40 min and subsequently with 30 ng cell free DNA (CfDNA) from AE17 cells for 4 h (right panels). Phosphorylated and total p65 as well as phosphorylated and total IKBa were measured by Western blot. (**E**–**G**) Tumors of vehicle- and karonudib-treated animals bearing AB1 or AE17 tumors were analyzed for central T subsets using flow cytometry. Total (**E**), Granzyme-B+, activated (**F**) and proliferating (**G**) CD8+ lymphocyte populations were quantified. (**H**) Activated CD11c+ MHCII+ dendritic cells were also determined in tumors. Data are presented as mean ± SEM, *n* = 5, * *p* < 0.05 compared to vehicle. The uncropped blots are shown in File S1.

Although we have recently shown that karonudib administration impedes experimental mesothelioma tumor progression and pleural fluid accumulation [12], we did not explore its potential impact on host’s immune responses. Here, we first focused on tumor-associated macrophages (TAMs), the predominant immune population in both mesothelioma tumors and mesothelioma-associated pleural effusion, which compromise T cell effectiveness [17]. We assumed that TAMs (which in the case of mesothelioma, are almost universally PD-L1+ and proliferating, [17]) might have an upregulated expression of MTH1 in response to oxidative stress posed by the tumor microenvironment. To elucidate this, we compared the MTH1 mRNA expression levels of macrophages isolated from murine tumors and pleural fluid to those residing in naïve lungs using real-time PCR (Figure 1A). MTH1 was found to be significantly elevated in both tumor and fluid macrophages compared to naïve ones (Figure 1A), implying that TAMs could thus be vulnerable to MTH1 inhibition. We therefore quantified macrophages in tumors and pleural fluid of karonudib- and vehicle-treated mice and evaluated their polarization. Interestingly, tumor macrophage infiltration was not affected by the treatment (Figure 1B), suggesting that MTH1 is rather inessential for their viability. Nevertheless, they presented enhanced M1 polarization (Figure 1C). Due to minimal pleural fluid accumulation in karonudib-treated animals (mean volume less than 50 μL), the observed significant reduction in total macrophages (Supplementary Figure S1A) and limited repolarization (Supplementary Figure S1A) were anticipated.

Having shown that karonudib does not compromise macrophage viability, we postulated that it might affect their function in an indirect manner. We have previously shown that mesothelioma cells secrete large DNA fragments [12]. Macrophages are known to respond to extracellular DNA stimuli through their TLR9 signaling [18]. We observed that the treatment of M2 macrophages with the extracellular DNA of karonudib-exposed mesothelioma cells activated the macrophage NFkB pathway by inducing *p*-ikBa phosphorylation (Figure 1D) and subsequent p65 activation (Figure 1D) and this effect was TLR9-mediated (Figure 1D).

We subsequently investigated the effects of karonudib on T cells, since new data on human mesothelioma support their TAM-mediated repression [17]. We observed that CD8 cell tumor infiltration was moderately enhanced by the treatment, since only AB1 tumors were significantly affected (Figure 1E). Nevertheless, CD8 T cell expansion was significantly enhanced in karonudib-treated tumors (Figure 1G), most probably as a consequence of the M1 macrophage polarization. T cell cytotoxicity was enhanced in cases of AB1 tumors (Figure 1F). We subsequently examined the possibility that karonudib directly affects T cells by comparing MTH1 mRNA levels among naïve and tumor-infiltrating lymphocytes. Since no impact was found (Supplementary Figure S1E), the observed effects of karonudib in CD8 T cells are most probably associated with karonudib’s effects on macrophages. Finally, karonudib enhanced MHCII expression by tumoral dendritic cells in the AB1 model (Figure 1H).

### 3.2. Karonudib Enhances Efficacy of Anti-PD-L1 Immunotherapy

**Figure 2:**
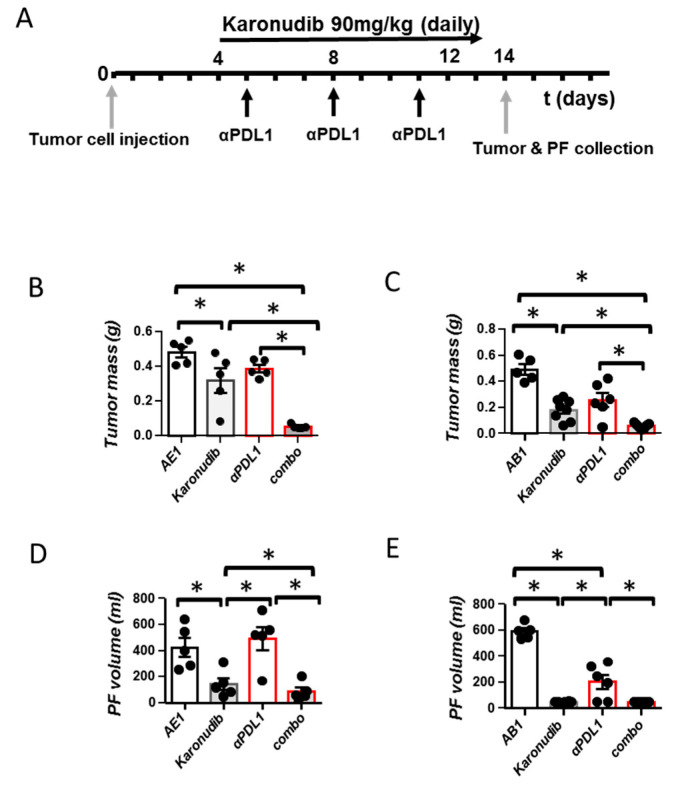
Karonudib enhances responses to anti-PD-L1 immunotherapy. (**A**) AE17 and AB1 cells were intrapleurally injected into syngeneic C57Bl/6 and Balb/c mice, respectively. Mice were administered with anti-PD-L1 (200 μg/dose i.p) every 3 days, karonudib (90 mg/kg body weight) twice bi-daily, anti-PD-L1 + karonudib (Combo) or respective vehicles (IgG isotype control and/or 10% *w*/*v* HPβCD). Fourteen days later, mice were sacrificed, mesothelioma tumors were excised and weighed (**B**,**C**) and pleural fluid was retrieved and quantified (**D**,**E**). Data are presented as mean ± SEM, *n* = 5–8, * *p* < 0.05 compared to indicated group.

The aforementioned results argue towards an immune-stimulating effect of karonudib and provide a rationale for its combination with immunotherapy. We, therefore, next investigated whether karonudib administration could enhance the efficacy of anti-PD-L1 treatment (following the regimen presented in Figure 2A) in experimental mesotheliomas. As seen in Figure 2B–E, dual therapy was more potent than single treatments in impeding tumor progression (Figure 2B,C). In addition to this, in AE17 tumor-bearing mice, dual therapy was more efficient in restricting pleural fluid accumulation compared with single regimens (Figure 2D). In AB1 mesotheliomas, since karonudib almost abolished fluid formation (<50 μL), no effect of the combined treatment was evident (Figure 2E).

### 3.3. Karonudib Enhances Anti-PD-L1 Immunotherapy’s Effects on CD8 T Cell Infiltration and Activation

**Figure 3:**
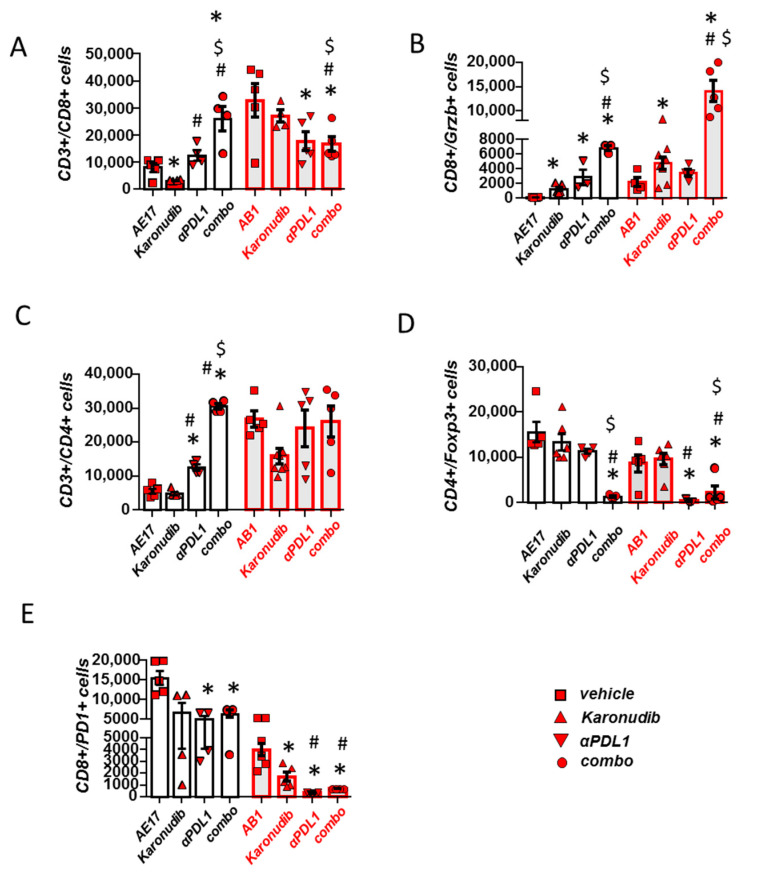
Karonudib reinforces the effects of anti-PD-L1 treatment on CD8 T cell infiltration and activation. (**A**) Tumors of vehicle-, karonudib-, anti-PD-L1- or combo-treated animals were analyzed for CD8 T cell infiltration and (**B**) activation. (**C**,**D**) CD4 and T regulatory cell tumor infiltration was also evaluated (**E**) “Suppressive” (PD1+) expression was evaluated among CD8+ lymphocytes. Data are presented as mean ± SEM, *n* = 5, * *p* < 0.05 compared to respective control, # *p* < 0.05 compared to Karonudib, $ *p* < 0.05 compared to anti-PDL1 group.

Analysis of tumor immune populations showed that T cell activation (Figure 3B) was enhanced upon combined treatment compared with administration of a single therapy. T cell infiltration was increased in case of AE17 mesotheliomas (Figure 3A,C). In addition to this, suppressive Treg populations were also significantly reduced with dual therapy compared to single ones (Figure 3D). No compensatory PD1 upregulation was observed among CD8 T cells in tumors of karonudib-treated animals, most probably because they are not directly affected by the treatment. PD1 expression was not further affected compared to the anti-PD-L1 regimen (Figure 3E).

In pleural fluid, combined treatment significantly upregulated CD8 T cell activation (Supplementary Figure S2B), although total CD8 numbers were reduced, most probably due to the substantial reduction in the effusion volume (Supplementary Figure S2A). Total CD4 populations were also reduced (Supplementary Figure S2C). Fluid Treg populations were not significantly modified by any manipulation (Supplementary Figure S2D). In the AB1 model, PD-1 expression was significantly reduced in CD8 populations in all treatment groups compared to controls but no difference was seen between combination therapy and monotherapies (Supplementary Figure S2E).

### 3.4. Combined MTH1 and PD-L1 Blockade Stimulates Macrophage M1 Polarization and DC Activation and Limits Suppressive MDSCs

**Figure 4:**
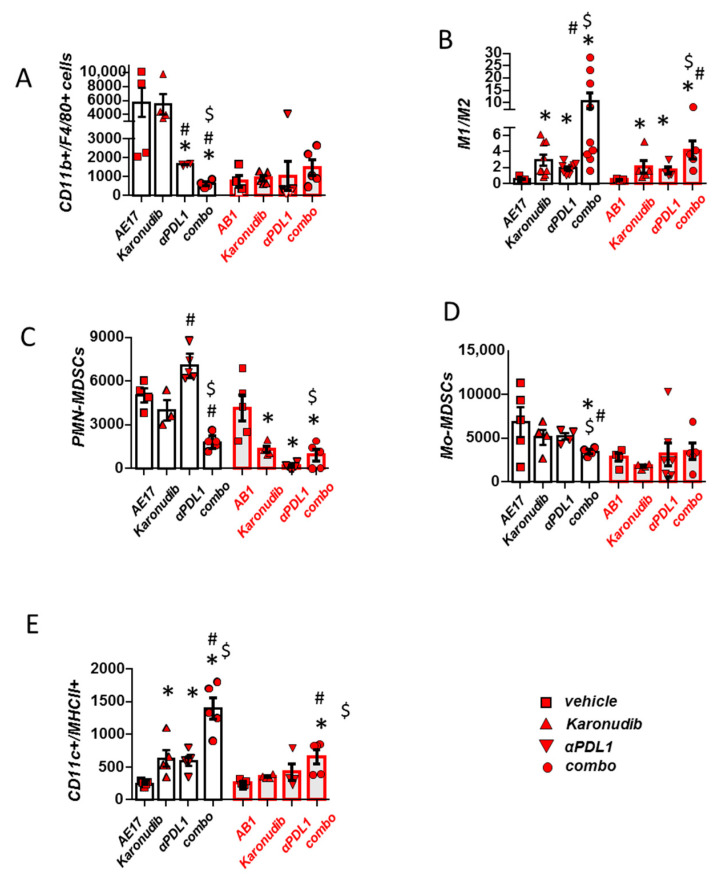
Combined MTH1 and PD-L1 blockade provokes macrophage M1 polarization and DC activation and limits suppressive MDSCs (**A**–**E**) Tumors of vehicle, karonudib, anti-PD-L1 or combo treated animals were analyzed for major myeloid populations using flow cytometry. (**A**,**B**) Total macrophage populations and macrophage polarization determined by IL12/IL10 expression ratio (indicative of M1/M2 polarization) were evaluated. (**C**,**D**) Tumor infiltration of myeloid-derived suppressor cells belonging to the monocytic (Mo) or polymophonuclear (PMN) fraction were enumerated. (**E**) Activation (MHCII+) of tumor dendritic cells (CD11c+) was also determined. Data are presented as mean ± SEM, *n* = 4–7, * *p* <0.05 compared to respective control, # *p* < 0.05 compared to Karonudib, $ *p* < 0.05 compared to anti-PDL1 group.

We finally evaluated the effects of dual therapy on myeloid populations. TAMs were massively reduced in AE17 tumors of mice receiving both regimens compared to single therapies, while in AB1 tumors remained unaffected (Figure 4A). Most importantly, combined treatment conferred a significant M1 polarization of macrophages in both models (Figure 4B). Of note, suppressive granulocytic MDSCs were also significantly reduced with combination treatment compared to monotherapies (Figure 4C). Monocytic MDSCs were significantly reduced in the AE17 model (Figure 4D). Finally, DC activation (MHCII presentation) was also significantly increased in both models (Figure 4E). Dual treatment enhanced M1 polarization and total macrophage numbers only in pleural fluid accompanying AE17 tumors (Supplementary Figure S3A,B). Dual treatment did not further reduce monocytic or granulocytic MDSCs, nor did it confer any additional effect on DC activation compared to monotherapies (Supplementary Figure S3C–E).

## 4. Discussion

The aim of this study was to investigate the immunoregulatory effects of MTH1 inhibition in mesothelioma and examine its potential to enhance the efficacy of immune checkpoint blockade. Our findings may be summed up as follows: (a) MTH1 inhibition confers an M1 phenotype among TAMs (through, at least in part, extracellular DNA/TLR9/NFkB signaling); (b) MTH1 inhibition stimulates CD8 T cell activation and tumor infiltration; and (c) treatment with an MTH1 inhibitor improves the efficacy of anti-PD-L1 treatment in experimental mesothelioma by favoring macrophage M1 polarization and DC activation and enhancing T cell tumor infiltration and activation triggered by immunotherapy.

We have previously shown that inhibition of MTH1 in mesothelioma cells hinders mesothelioma growth by making tumor cells and tumor endothelium vulnerable to microenvironments experiencing oxidative stress [12]. Given that oxidative stress also regulates anti-tumor immune responses [19,20,21,22] and DNA repair/damaging agents exert immunomodulatory actions [11,23,24], we also examined whether MTH1 is involved in the regulation of tumor immunity. We first focused on ΤAΜs since they are the most abundant immune population of both mesothelioma tumors and accompanying pleural fluid [17]. Although TAMs were found to overexpress MTH1 compared to those residing in the normal pleura, implying that they could be directly targeted by the regimen, they were not eliminated by karonudib. This implies a non-essential role of MTH1 in TAMs’ survival, which could be associated with the MTH1-independent 8-oxodGTPase activity [25] that is present in macrophages and compensates for MTH1 de-activation. Nevertheless, MTH1 inhibition enhanced M1 polarization, which is vital for effective anti-mesothelioma immune responses and tumor [16,26,27]. We also observed enhanced CD8 T cell activation and infiltration in tumors of karonudib-treated groups, which is a common sequelae of TAM reprogramming towards the M1 phenotype [15,16,27,28,29]. Of note, we herein show that karonudib favors the M1 polarization of TAMs through heterotypic signaling between mesothelioma tumor cells and macrophages. Cell-free DNA from karonudib-treated mesothelioma cells was able to activate NFkB (previously shown to mediate the transcription of M1-associated genes) [30] through Tthe LR9 receptor of macrophages, whereas the respective cell-free DNA from vehicle-treated ones could not. This could be associated with high levels of oxo-d-G in the DNA fragments of karonudib-treated tumor cells compared to vehicle-treated ones [12].

Since MTH1 inhibition was found to alleviate the suppressive properties of TAMs and preserve T cell fitness, we subsequently investigated its potential to enhance the efficacy of standard anti-PD-L1 immunotherapy in vivo. Research is increasingly focusing on combinations of novel DNA repair inhibitors with immunotherapy [31] based on the rationale that targeting DNA repair mechanisms could lead to increased mutational burden, enhance cancer immunogenicity and potentiate immune checkpoint blockade responses [11,23,32,33]. To this end, MTH1 inhibition can be superior to DNA repair targeting agents, since it is vital for the survival of tumor cells without affecting normal cells’ viability, a condition known as phenotypic lethality [34,35]. The most obvious benefit of this feature is the broad spectrum of cancer types in which such an approach might be applicable, followed by the limitation of side effects, the minimum risk of resistance emerging [34,35] and the preservation of immunity [36]. In our hands, karonudib administration, apart from reversing the skewing of innate immune populations, did not compromise T cell functions. The importance of this finding lies in the fact that dysfunctional T cells are the main impediment to the effectiveness of immunotherapy [37], including mesothelioma [38]. Not surprisingly then, the dual administration of karonudib and anti-PD-L1 was more potent in halting mesothelioma progression by triggering more profound CD8+ T cell and DC activation, encouraging M1 polarization and reducing Treg suppressive actions compared to single therapies.

## 5. Conclusions

Taken together, our results suggest that the dual inhibition of MTH1 and PD-L1 is a vital strategy against mesothelioma progression and supports the significance of clinical trials to test this possibility.

## Data Availability

The datasets used and/or analyzed during the current study are available from the corresponding author on reasonable request.

## Supplementary Materials

The following supporting information can be downloaded at: https://www.mdpi.com/article/10.3390/cancers15204962/s1, Figure S1: Effects of Karonudib on macrophages and lymphocytes of mesothelioma-associated pleural effusions. Figure S2: Effects of Karonudib, anti-PDL1 and combined treatment on lymphocytes of mesothelioma-associated pleural effusion. Figure S3: Effects of Karonudib, anti-PDL1 and combined treatment on central monocytic populations of mesothelioma-associated pleural effusion. File S1: Western blots information.
