# Supplementary material for: MTH1 Inhibition Alleviates Immune Suppression and Enhances the Efficacy of Anti-PD-L1 Immunotherapy in Experimental Mesothelioma
Source: Cancers (Basel). 2023 Oct 12;15(20):4962. doi: 10.3390/cancers15204962 (PMC10605650; doi:10.3390/cancers15204962)
Supplement: Supplementary file 1 [file cancers-15-04962-s001.zip › cancers-2510909-supplementary.pdf]

Supplementary Data

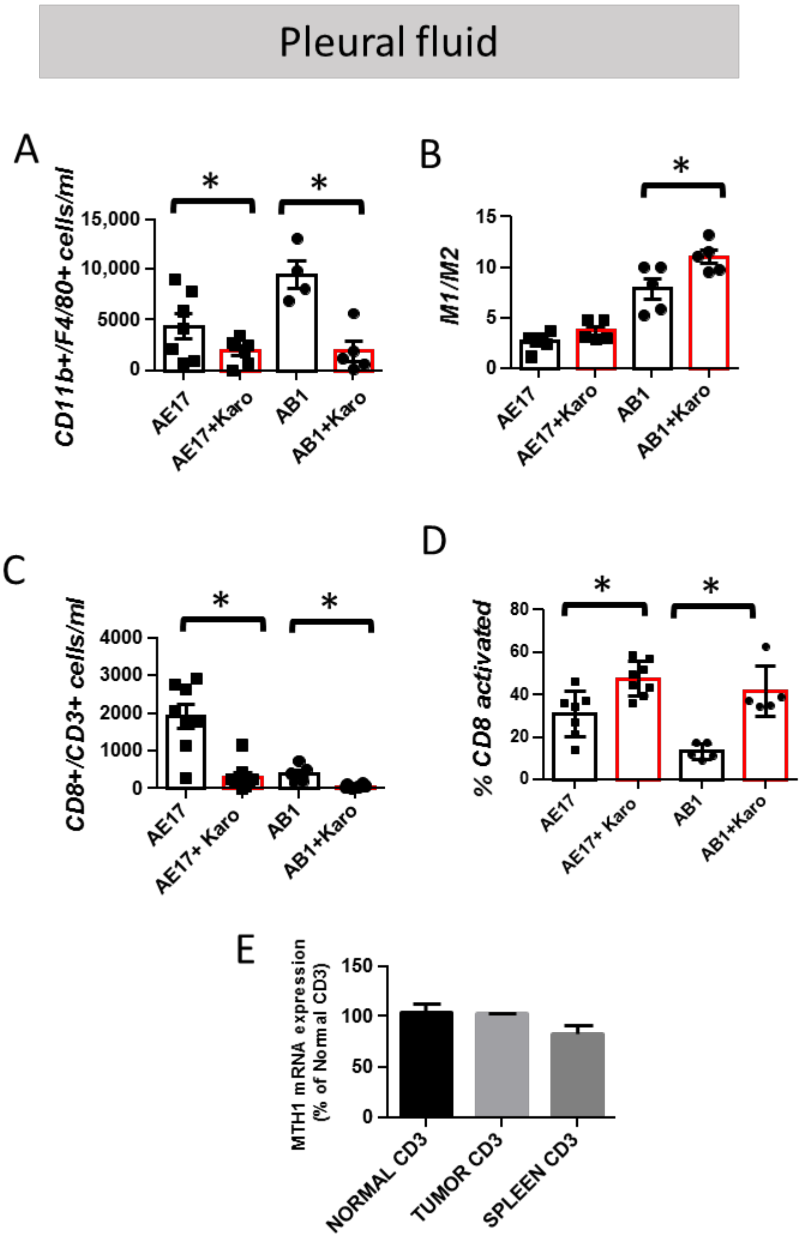

Supplementary Figure S1. Effects of Karonudib on macrophages and lymphocytes of mesothelioma-associated pleural effusions. AB1 and AE17 cells were intrapleurally injected into syngeneic Balb/c and C57Bl/6 mice, respectively. Mice were subsequently treated with vehicle or Karonudib as aforementioned. Pleural fluid of vehicle and Karonudib treated animals was analyzed by flow cytometry for (A,B) total macrophages (CD11b+ F4/80+) and their IL12/IL10 expression ratio (indicative of M1/M2 polarization). Data are presented as mean±SEM, n=5-8, *p<0.05 compared to vehicle. (C,D) Total and activated pleural fluid CD8+ lymphocyte populations were quantified. Data are presented as mean±SEM, n=5, *p<0.05 compared to vehicle. (E) T cells from mesothelioma tumors, accompanying pleural fluid and spleen were isolated by anti-CD3 magnetic beads and Mth-1 mRNA expression was determined by

Real-time PCR. Data presented as mean±SEM, n=3,*p<0.05 compared to 'normal' lymphocytes.

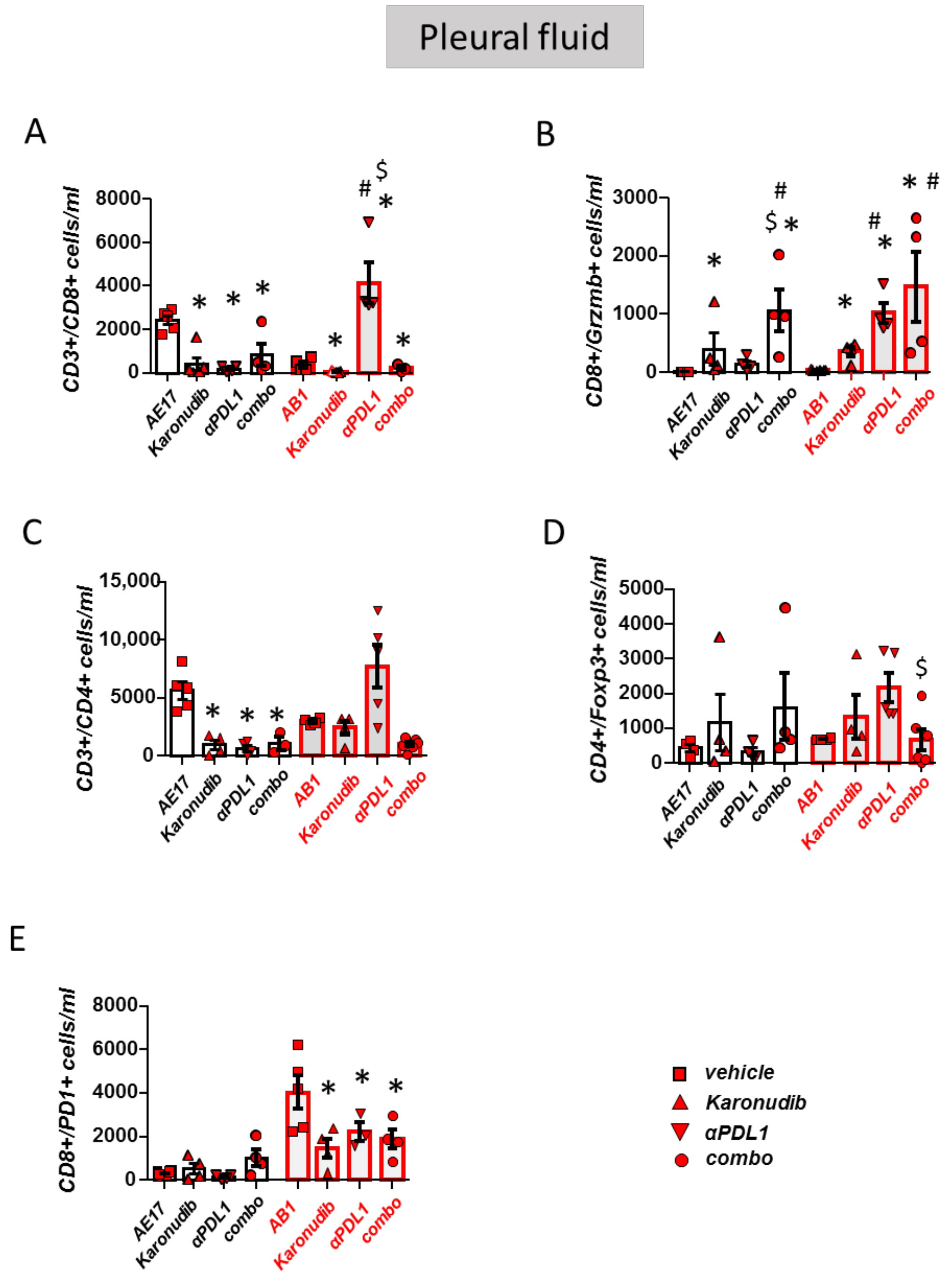

Supplementary Figure S2. Effects of Karonudib, anti-PDL1 and combined treatment on central monocytic populations in mesothelioma associated pleural effusion. Pleural cells of vehicle, Karonudib, anti-PDL1 or combo treated animals were analyzed for major myeloid populations using flow cytometry. (A, B) Total macrophage populations and their polarization was determined using IL12/IL10 expression ratio (indicative of M1/M2 polarization). (C, D) Myeloid derived suppressor

cells belonging to the Monocytic (C) or Polymophonuclear (PMN) fraction were enumerated. (E) Activation (MHCII+) of tumor dendritic cells (CD11c+) was also determined. Data are presented as mean±SEM, n=5, *p<0.05 compared to indicated group.

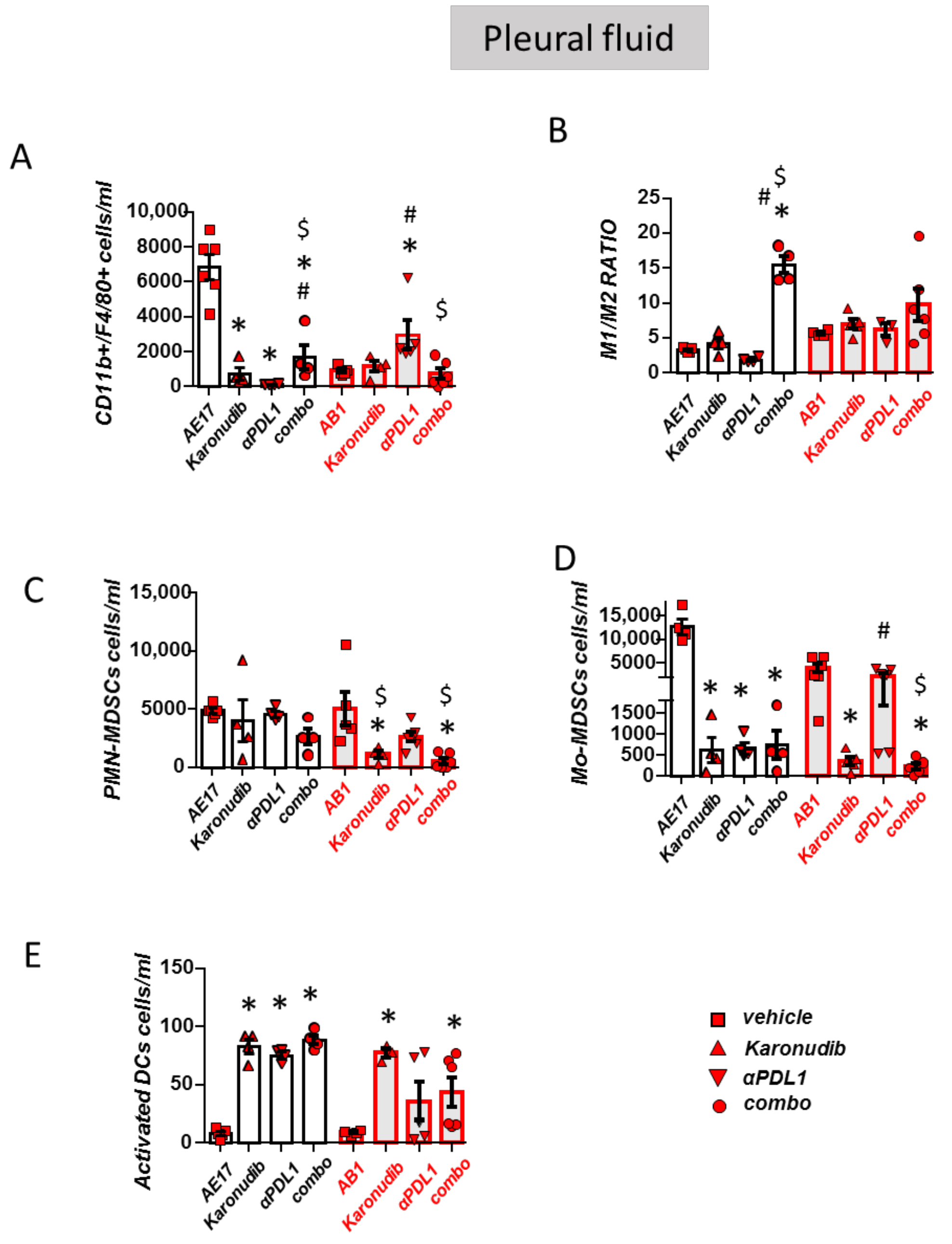

Supplementary Figure S3. Effects of Karonudib, anti-PDL1 and combined treatment on lymphocytes in mesothelioma associated pleural effusion. (A) Pleural cells of vehicle, Karonudib, anti-PDL1 or combo treated animals were analyzed for total and (B) activated T cell populations. (C) Total CD4 T cell numbers were also determined as well as suppressive T regulatory cell populations (D). (E) “Suppressive” (PD1+) expression was evaluated among CD8+ lymphocytes. Data are presented as mean±SEM, n=5, *p<0.05 compared to indicated groups.
