# Supplementary figures and images for: MTH1 Inhibition Alleviates Immune Suppression and Enhances the Efficacy of Anti-PD-L1 Immunotherapy in Experimental Mesothelioma
Source: Cancers (Basel). 2023 Oct 12;15(20):4962. doi: 10.3390/cancers15204962 (PMC10605650; doi:10.3390/cancers15204962)

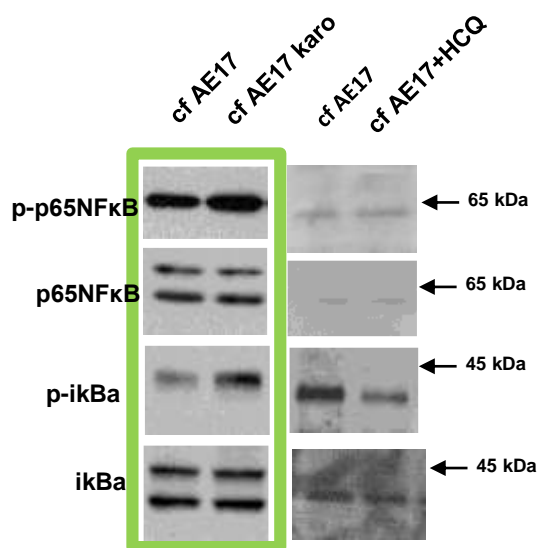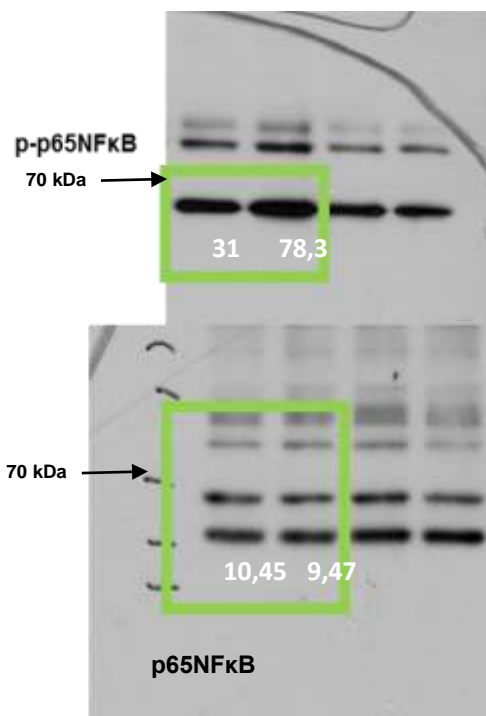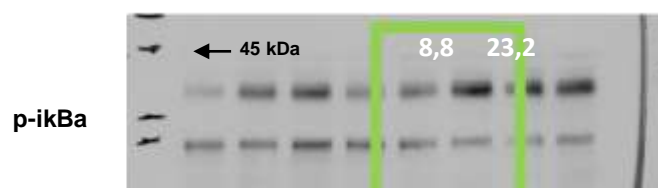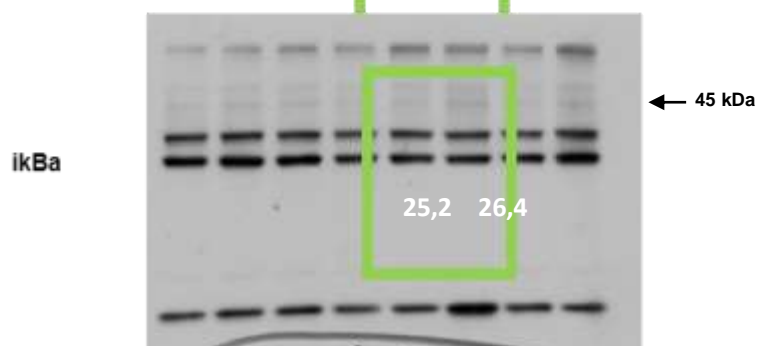

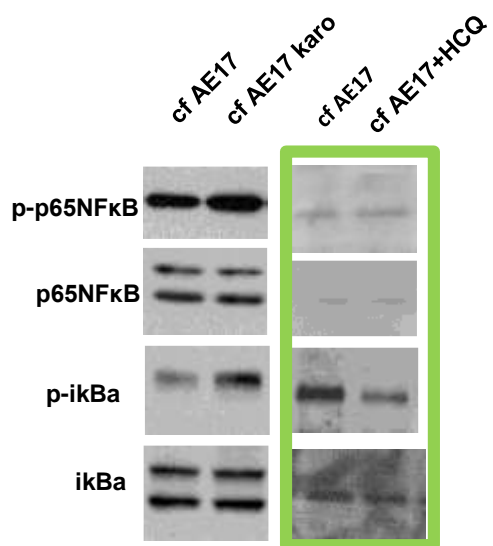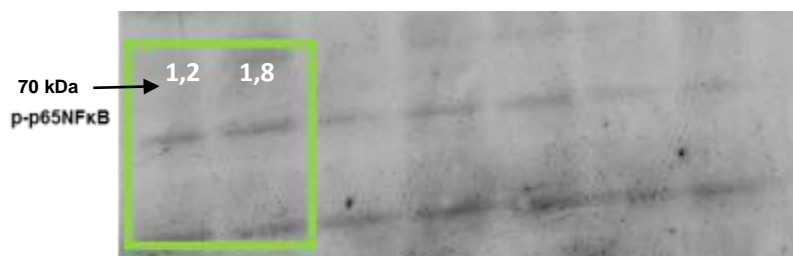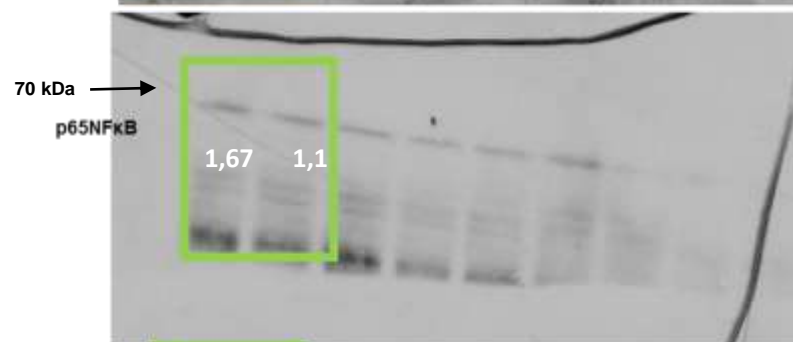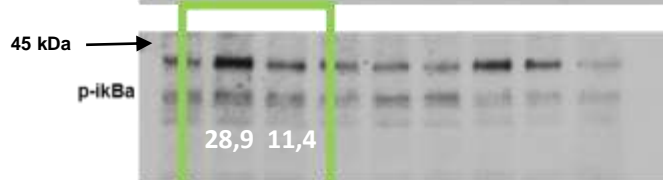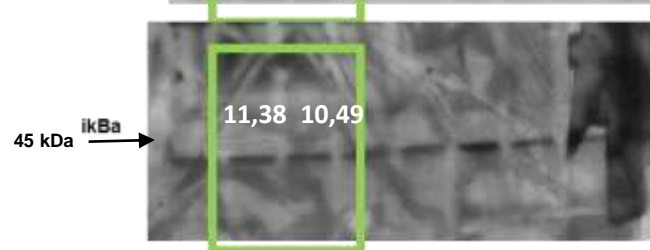

Supplement: Supplementary file 1 [file cancers-15-04962-s001.zip › supplementary file S1 ORIGINAL BLOTS.pdf]
